# Antibiotics and Therapeutic Agent Prescription in COVID-19 Management

**DOI:** 10.3390/antibiotics11040423

**Published:** 2022-03-22

**Authors:** Souheil Zayet, Timothée Klopfenstein

**Affiliations:** Infectious Disease Department, Nord Franche-Comté Hospital, 90400 Trevenans, France

During the coronavirus disease 2019 (COVID-19) pandemic, only few therapeutic options have been approved for the treatment of COVID-19 with substantial evidence. The spectrum of effective therapies to treat moderate-to-severe COVID-19 forms or prevent disease progression is growing and evolving rapidly. Several trials to assess the potential effectiveness of these therapies in hospitalized adults and outpatients, but also in specific populations such as children and pregnant women, are ongoing. Until now, only corticosteroids and interleukin 6 (IL-6) receptor antagonists such as tocilizumab were retained as proven therapies for COVID-19 in RECOVERY study results from June 2020 [1] and February 2021 [2], and should be administrated with heparin on the basis of risk/benefit according to disease severity. However, some physicians and doctors are still prescribing inappropriate treatment for COVID-19 patients. 

This Special Issue includes full research articles and perspectives focused on therapeutic agent prescription in COVID-19 management. 

Among the first type of contributions, Alaa Thabet Hassan and coworkers [3] concluded in a prospective study including 123 severe COVID-19 pneumonia patients that the use of combined methylprednisolone and low molecular weight heparin (LMWH) as therapeutic enoxaparin, according to a flexible protocol (Appendix A), led to prevent disease complications and improved clinical outcome. 

The beneficial effects of anticoagulants on the clinical outcome of COVID-19 patients were also the focus of the contribution of Zubia Jamil and coworkers [4]. The authors reported that both anticoagulant formulations; enoxaparin (therapeutic and prophylactic doses) and heparin (prophylactic dose), were associated with improving survival among moderate-to-severe COVID-19 patients, using mortality at 30-day as a primary endpoint. In a second contribution [5], the same author and coworkers compared the effects of different formulations and doses of steroids on the 30 day in-hospital clinical outcome of 557 patients with severe acute respiratory syndrome coronavirus-2 (SARS-CoV-2). They suggested that low-dose dexamethasone (6 mg/day) is more effective than high-dose dexamethasone (>6 mg/day) and methylprednisolone (500 mg/day) given for 10 days in improving the survival outcome of severe COVID-19 patients. In a further clinical study control including 184 patients [6], the predictive value of cell blood count (CBC) parameters as biomarkers of acute pulmonary embolism (aPE) in patients with SARS-CoV-2 infection was investigated by Alessio Strazzulla and coworkers. The results revealed that COVID-19 patients with aPE had higher neutrophil-to-lymphocyte ratio, platelet-to-lymphocyte ratio, and neutrophil and lymphocyte counts than patients without aPE with a significant difference. Indeed, neutrophil and lymphocyte counts were both associated with diagnostic of aPE while no CBC parameters were associated with mortality at day 7.

Antibiotics have been used extensively during COVID-19 pandemic as a protective measure against secondary bacterial co-infection or superinfections [7]. However, this overprescription might be contributing to antimicrobial resistance emerging. Ragaey A. Eid and coworkers [8] performed Mpro enzyme assays (In vitro and in silico) to investigate the antiviral potential of ceftazidime and cefepime in 370 moderate-to-severe COVID-19 patients. They concluded that both antibiotics (1000 mg twice daily for five days, for each antimicrobial drug) showed very good inhibitory activity towards SARS CoV-20’s M^pro^, with IC_50_ values of 1.81 µM and 8.53 µM, respectively, without any adverse effects. Antimicrobial overprescription was studied during presumed urinary tract infection (UTI) in patients admitted with COVID-19. Johan Van Laethem and coworkers [9] performed a quantitative and qualitative assessment of UTI diagnoses and antimicrobial drug prescriptions for UTI diagnoses in 622 confirmed COVID-19 patients. This evaluation revealed that of the 79 UTI diagnoses, 61% were classified as probable overdiagnosis related to the COVID-19 hospitalizations and were not appropriate. The authors also concluded that associated factors with UTI overdiagnosis had been made by physicians who were unfamiliar working in an internal medicine department, urinary incontinence, mechanical ventilation and female sex. 

The contribution of Tito Ramírez-Lozada and coworkers [10] evaluated the prescription of antibiotics in 50 pregnant patients infected with SARS-CoV-2, especially during the first trimester of pregnancy, considered as a period with state of immune tolerance with predisposition to viral infection. The authors concluded that antibiotics in pregnant or lactating women should not be considered part of the initial treatment of SARS-CoV-2 infection since the vertical transmission of these drugs and microorganisms resistant to them from mother to child harms the development and succession of the infant’s gut microbiota. They also recommended performing several and continuous representative cultures with antimicrobial testing susceptibility.

Batool Butt and coworkers [11] were interested in another vulnerable population; COVID-19 hemodialysis patients. The authors concluded that using Remdisivir, the first antiviral drug approved by the Food and Drug Administration (FDA) in 2020 [12], as an RNA-dependent RNA polymerase (RdRp) inhibitor, (at the dose 100mg before hemodialysis), could shorten the recovery time for dialysis patients (n = 83) if taken within 48 hours before the onset of symptoms, with an excellent safety. Remdesivir administration might probably reduce the duration of hospitalization (*p* = 0.03), with an earlier negative COVID-19 RT-PCR (*p* = 0.001) in survivors who received Remdesivir within 48 h of diagnosis.

Effective oral antiviral agents against SARS-CoV-2 have recently been developed to treat COVID-19, block SARS-CoV-2 transmission, and prevent progression to severe illness. In this item, Ching-Chi Lee [13] and Yuan-Pin Hung [14] contributed to this Special Issue with two papers as perspectives. The first focus on Molnupiravir (formerly EIDD-2801), a prodrug of beta-D-N4-hydroxycytidine and a RdRp inhibitor, with a direct-acting activity against SARS-CoV-2 (in vivo, and in the animal model) [13]. Moreover, Molnupiravir seems to be effective at reducing the nasopharyngeal viral load with a good safety and tolerability profile in COVID-19 patients receiving short-course (at 5 days). An updated phase-III trial revealed that Molnupiravir significantly reduced the risk of hospitalization and/or death in mild-to-moderate COVID-19 patients. The second contribution focuses on the novel oral Nirmatrelvir/ritonavir (Paxlovid™), a SARS-CoV-2 main protease (Mpro) inhibitor (also known as SARS-CoV-2 3CL protease inhibitor) therapy, which has recently been approved by the FDA, with the particularity to be efficient against the SARS-CoV-2 omicron variant [14,15]. Based on the preliminary results of the EPIC-HR trial, early administration of oral nirmatrelvir/ritonavir therapy may effectively decrease the risk of COVID-19-related hospitalizations or death in outpatients (mild-to-moderate COVID-19 within five days of symptom onset).

This Special Issue collects multidisciplinary researches in view of the health emergency and the use of therapeutic agents with high uncertainty and potential for harm in order to improve treatment recommendations during COVID-19. The contributions collected in This Special Issue constitute a valuable knowledge reservoir for scientists to choose effective therapeutic agents against SARS-CoV-2 and to the indiscriminate use of antibiotics in COVID-19 patients.

## Appendix A

**In Alaa Thabet Hassan et al.; anticoagulation was prescribed as followed (the Flexible Protocol):** All patients with severe COVID-19 pneumonia received at least Enoxaparin 1 mg/kg twice daily, up to 80 mg twice daily; to patients with thrombocytopenia, Fondaparinux was administered at a dose of 5 mg SC for patients < 50 kg and 7.5 mg for patients > 50 kg. After clinical and radiological improvement and reduction of FiO_2_, the dose was titrated down by 25%, and then by 50%. The dose of Enoxaparin was decreased to 40 mg SC, once daily, and the dose of Fondaparinux was decreased to 25 mg once daily when O_2_ requirements become 6 L or less; finally, on discharge, patients were discharged on rivaroxaban 10 mg, or any other NOACS, once daily for 1 month.
